# The effects of 14-week betaine supplementation on endocrine markers, body composition and anthropometrics in professional youth soccer players: a double blind, randomized, placebo-controlled trial

**DOI:** 10.1186/s12970-021-00417-5

**Published:** 2021-03-04

**Authors:** Hadi Nobari, Mehdi Kargarfard, Vazgen Minasian, Jason M. Cholewa, Jorge Pérez-Gómez

**Affiliations:** 1 Department of Exercise Physiology, Faculty of Sport Sciences, University of Isfahan, Isfahan, Iran; 2 Department of Exercise Physiology, College of Health Sciences, University of Lynchburg, Lynchburg, VA 24501 USA; 3 HEME Research Group, Faculty of Sport Sciences, University of Extremadura, Cáceres, Spain

**Keywords:** Nonfunctional over-reaching, Youth sports, Lean mass, Football, Testosterone

## Abstract

**Objective:**

Betaine supplementation may enhance body composition outcomes when supplemented chronically during an exercise program. The purpose of this study was to evaluate the effect of betaine supplementation on development-related hormones, body composition, and anthropometrics in professional youth soccer players during a competitive season.

**Methods:**

Twenty-nine players (age, 15.45 ± 0.25 years) were matched based upon position and then randomly assigned to a betaine group (2 g/day; *n* = 14, BG) or placebo group (PG, *n* = 15). All subjects participated in team practices, conditioning, and games. If a subject did not participate in a game, a conditioning protocol was used to ensure workload was standardized throughout the 14-week season. Growth hormone (GH), insulin-like growth factor-1 (IGF-1), testosterone, cortisol, height, weight, and body composition were assessed at pre-season (P1), mid-season (P2) and post-season (P3). Anthropometric variables were also measured following a one-year follow-up (F).

**Results:**

Significant (*p* < 0.05) group x time interactions were found for testosterone and testosterone to cortisol ratio (T/C). Both variables were greater in BG at P2 and P3 compared to P1, however, the testosterone was less in the PG at P3 compared to P2. There was no significant group by time interactions for GH, IGF-1, lean body mass, or body fat. There was a significant (*p* < 0.05) group x time interaction in height and weight at F, with the greater increases in BG compared to PG.

**Conclusion:**

Betaine supplementation increased testosterone levels and T/C ratio in youth professional soccer players during a competitive season. Betaine supplementation had no negative effects on growth (height and weight) and may attenuate reductions in testosterone due to intense training during puberty.

**Supplementary Information:**

The online version contains supplementary material available at 10.1186/s12970-021-00417-5.

## Introduction

Soccer is undoubtedly the most popular sport in the world. Soccer is a high intensity intermittent sport [1], and players usually run about 10 km during a game, of which 2–3 km is comprised of high-speed running and about 0.5 km is ran at maximum speed [2, 3]. Soccer also requires ballistic concentric and eccentric movements throughout the game, including acceleration, deceleration, change of direction, tackling, jumping and shooting [4]. These actions result in a diverse metabolic and physiological stress to support running, sprinting, stopping, rotation, and jumping [5].

Many studies have examined the relationship between anthropometry, muscular performance and soccer specific physical performance (i.e., sprint, repeated sprint ability, vertical jump, etc.). These studies demonstrated a strong relationship between body composition (high levels of lean mass and low fat mass) with vertical jump performance and repeated sprint ability in both elite [6] and youth soccer players [7]. In addition to leaner body compositions, metrics of lower body strength are also strongly associated with acceleration, sprinting, and jumping performance in youth soccer players [8]. Given these relationships, dietary and training interventions that improve the power to mass ratio by increasing lower body strength and/or reducing adiposity should lead to large improvements in the physical performance attributes of youth soccer players [9].

Soccer players accumulate fatigue as a season progresses, and when the volume of training/competition increases in conjunction with insufficient recovery, players may enter into a nonfunctional over-reaching state (NFO) marked by decrements in performance [10]. In addition to monitoring performance, the routine monitoring of endocrine hormones may be used as biomarkers of physiological stress that may affect recovery and performance throughout the season [11]. In particular, changes in growth hormone (GH), testosterone, cortisol, and the testosterone/cortisol ratio (T/C) have all been reported as valid biomarkers that may indicate NFO in soccer players [10–14].

Dietary and ergogenic supplements have been added as an intervention in athletes’ programs to achieve faster adaptation and manage fatigue from exercise. Trimethyl-glycine (betaine) is a zwitterionic amino acid found in green leafy vegetables and betaine anhydrous, which is available as a dietary supplement, is a biproduct of sugar extraction from beet roots. Six to 10 weeks of betaine supplementation along with resistance training has been shown to decrease fat mass [15, 16] and increase lean mass in previous studies [15]. Up to 2 weeks of betaine supplementation has shown improvements in upper and lower body force and power output [17], power output during cycle sprinting [18], strength [19], and muscular endurance [20]. These results provide a rationale whereby betaine supplementation may improve soccer performance by enhancing the power to mass ratio.

Betaine supplementation may also enhance recovery and delay the onset of NFO during a season. Betaine increased GH, insulin growth factor-1 (IGF-1), and protein kinase B (Akt) phosphorylation [21] in young adults. Betaine also agonized IGF-1 receptors and promoted myosin heavy chain protein synthesis in vivo [22]. Interlukin-1 beta (IL-1ß) accumulation leads to intramuscular inflammation following eccentric exercise [23], and betaine supplementation has been shown to dampen the inflammatory response by lowering the secretion of IL-1ß in addition to several other pro-inflammatory cytokines [24]. Additionally, 6 weeks of betaine supplementation attenuated reductions in vertical jump performance with high-volume resistance training [15]. Whether these mechanisms translate to meaningful changes in hormone concentrations, body composition, or performance in competitive soccer players needs to be elucidated.

Therefore, the primary aim of this study was to investigate the effects of betaine supplementation on hormonal status, anthropometrics, and body composition during a 14-week competitive season. A secondary purpose was to measure if any changes found in growth are sustainable, by performing a one-year follow up. We hypothesized that betaine supplementation will reduce indices of NFO and result in greater improvements in measures of body composition during a competitive season.

## Materials and methods

### Selection of study groups

The participants (*n* = 29) were professional youth soccer players under 16 (U16) in the Sports Club competing in the Iranian Youth Premier League. The inclusion criteria were: (i) Attend all training sessions, (ii) no vitamins or any other supplement should be taken during the intervention, (iii) no cross-training during the study, (iv) no supplement use for at least one year prior to the study. To achieve this goal, a demographic questionnaire and sport-medical records were used. The exclusion criteria were: (i) Incurring any injury that prevented participation in training or games. As a result of this criteria, one player was removed from the study in the betaine group (BG). Because of the differences in energy systems used in different soccer positions, we divided the team into five general categories: Goalkeepers (*n* = 2), defenders (*n* = 8), halfback (n = 8), winger (*n* = 6), and forwards (*n* = 5). Participants were then randomly, within positions. Randomization was based on participant field positions and accomplished via a computer-generated randomized table, creating a betaine group (BG: *n* = 14) and placebo group (PG: *n* = 15). No significant differences were observed between groups at baseline (Table 1). All players, along with their parents, were notified of the potential risks and benefits of participating in the research before the study began. Subjects, as well as their parents, signed a consent letter to participate in the project. Prior to the start of the study, the Ethics Committee of the University of Isfahan approved the study (IR.UI.REC.1398.102), and the recommendations of Human Ethics in Research were followed by the Helsinki Declaration.

**Table 1:** Descriptive characteristics of BG (*n* = 14) or PG (*n* = 15) in the soccer players U16, separately

Variables	Groups	***Mean ± SD***	***Minimum***	***Maximum***
Height (cm)	*BG*	172.1 ± 2.3	170	177
	*PG*	174.2 ± 4	168	183
Weight (kg)	*BG*	59.2 ± 4.8	53	68.5
	*PG*	65.8 ± 7.0	56.5	79.9
Sitting height (cm)	*BG*	92.7 ± 1.4	90.0	96.5
	*PG*	93.9 ± 3.3	89.0	99.0
PHV (years)	*BG*	13.6 ± 0.2	13.2	14.0
	*PG*	13.4 ± 0.5	12.5	14.1
Maturity Offsets (years)	*BG*	1.8 ± 0.2	1.5	2.3
	*PG*	2.1 ± 0.4	1.3	2.7
Age (years)	*BG*	15.4 ± 0.3	15.0	15.8
	*PG*	15.5 ± 0.2	15.0	15.7
Experience (years)	*BG*	6.6 ± 1.5	4	9
	*PG*	6 ± 1.6	4	9
BMR (kcal/day)	*BG*	1628.9 ± 72.9	1543.8	1771.1
	*PG*	1730.3 ± 109.3	1581.7	1942.3
Total calories (kcal/day)	*BG*	2524.7 ± 112.9	2392.8	2745.2
	*PG*	2681.9 ± 169.5	2451.7	3010.6

*SD* Standard deviation; *PG* Placebo group; *BG* Betaine group; *PHV* Peak height velocity; *BMR* Basal metabolic rate

### Experimental approach to the problem

The present study was a quasi-experimental design with pre-test, mid-test, and post-test. Participants consumed 1 capsule, twice daily, with 300 ml water approximately 2 h prior to training and one-hour post training. The BG ingested betaine and the PG consumed a flour. Both groups took a total of 2 g/day (1000 mg per capsule), and the capsules were the same shape, size and white color. The researcher contacted the players on a daily basis to ensure compliance, and parents monitored the process of regular consumption. Anthropometric measurements, body composition, and blood tests were conducted at pre-season (P1), mid- season (P2) and post-season (P3), and were performed 48 h apart from the last training. Participants recorded their nutrition for three full days and delivered it to the researchers at each testing time-point. All players participated in the same standardized training sessions during the study. In order to match the load of the weekly workouts, players who did not participate in competitions or participated in only one half were to perform a post-match, small sided game, individual training, or friendly competition. Subjects presented individual wellness questionnaires before the training sessions and reported internal training load via the session rating perceived exertion (sRPE) 30 min after the end of each training session. To observe the effect of 14 weeks of betaine supplementation on physical growth of the athletes, anthropometric measurements were repeated via the same methods and instruments under the supervision of researchers at the club academy 1 year following the cessation of the supplementation.

### Procedures

#### Blood analysis

Subjects reported to the Alzahra Hospital’s lab for blood sampling following a 12 h fasting, and at least 48 h following the last training session. To account for circadian rhythms, 10 cc of blood were collected from the antecubital vein before 9 am. Samples were immediately centrifuged, the serum was separated, and then assayed on the same day to measure the GH, testosterone, cortisol, and IGF-1. To measure GH and IGF-1 the chemiluminescence method (ICMA) with IMMULITE 2000xpi Systems (SIMENS Germany, Specifications of GH kits; REF: L2KGRH2; lot 191) were used. The analytical sensitivity of the kit is 0.01 ng/mL. The average inter-assay coefficients of variability (CVs) were 3.76% and the mean concentrations were 7.3 ng /mL. For IGF-1 (kit REF: L2KIGF2 and lot: 575) the limit was 13.3 ng/mL. The average inter-assay CVs were 4.4% and the mean concentration was 379.83 ng /mL.

To measure cortisol and testosterone the ICMA with ADVIA (Centaure XPT System, SIMENS Germany, specifications of cortisol kits; REF: 04610138; lot: 019321) were used and the analytical sensitivity of assay ranges was 0.20–75 μg/dL (5.5–2069 nmol/L). The average inter- and intra assay CVs were 3.29 and 3.64%, respectively, for the mean concentrations of 17.67 μg/dL. For testosterone (REF: 05476206; lot: 180025) the analytical sensitivity of assay ranges was 10–1500 ng/dL (0.35–52.1 nmol/L). The average inter- and intra assay CVs were 4.05 and 3.7%, respectively, for the mean concentration of 567.17 ng/mL.

#### Anthropometric and body composition

Standing height was measured via standard anthropometry with subjects standing barefoot against a Seca model 213 Stadiometer (Seca 213, Germany). To measure seated height, subjects sat on a 50 cm box, facing forwards with their lower back as close to the Stadiometer as possible and their head and necks in an anatomically neutral position. Height was measured from the highest point of the head to the base of the ischium (top of the box). Weight was measured with subjects dressed only in athletic shorts on a scale (Seca 813, UK) with an accuracy of 0.1 kg.

To determine the maturity offset and age at peak height velocity of the subjects, the formula was used [25] as follows: Maturity offset = − 9.236 + 0.0002708 (leg length × sitting height) − 0.001663 (age × leg length) + 0.007216 (age × sitting height) + 0.02292 (weight by height ratio), where correlation coefficient (R) = 0.94, r-squared (R^2^) = 0.891, and standard error of the estimate (SEE) = 0.592, also, the following formula was used to obtain leg length: Standing Height (cm) - Sitting height (cm) [26, 27].

Skin fold calipers (Lafayette Instrument Company, Lafayette, IN, USA) were calibrated according to manufacturer instructions to an accuracy of 0.1 mm and skinfold measurements were obtained from the chest, abdominal, thigh, triceps, subscapular, suprailiac and midaxillary and used to calculated body density via Jackson and Pollock method [28]. Body fat percentage (BF%) was then estimated via Brozek’s formula [28], similar to previous studies in adolescent soccer players [26, 27, 29]. All measurements were performed twice on the right side of the body; the final score was recorded with the mean of two measurements. To reduce measurement error, if the technical measurement error was high (> 5%), the subcutaneous fat measurement was performed again and the median three repetitions were used for analysis [26, 30]. All measurements were performed by an expert with 5 years of background in this area. All anthropometric and body composition measurements were taken in the morning (08:00–11:00 h) [31, 32] immediately following the blood collection.

#### Control of food intake

One week before the start of the study, athletes were asked to record and deliver a whole day of total nutrients (carbohydrate, fat and protein). Using the Harris Benedict method [33], the basal metabolism of each subject was calculated and according to weekly sessions with the criterion of medium exercise, the basal metabolic rate was multiplied by 1.55 and the total calorie requirement of each athlete was calculated [34]. A nutritionist outside of the study then created a list of approximate calorie intake of all Iranian native foods, instructed the subjects how to obtain their individual necessary caloric intake, and distributed the recommendations in writing to the subjects. 72 h prior to each of the three stages of blood testing, daily dietary intakes of the subjects were measured and total calorie intake was calculated using Nutrition 4 version 3.5.2 software made in Iran. Daily reports by subjects showed that there was no significant difference between the two groups for nutrients consumed at any time point (Table 2).

**Table 2:** The analysis of the average daily caloric and macronutrient intake

Nutrient	Groups	Pre-Season	Mid- Season	Post- Season	MET (***p***)
		M ± SD	M ± SD	M ± SD	
Kilocalories (kcals/day)	BG	2535.6 ± 134.6	2570.7 ± 125.5	2550.8 ± 159.9	0.85
	PG	2600 ± 214.6	2559.8 ± 199.2	2561.2 ± 183.4	
Carbohydrate (g)	BG	359.6 ± 19.0	366.5 ± 18.0	365.1 ± 22.4	0.95
	PG	370 ± 30.7	364.8 ± 28.4	366.1 ± 25.8	
Protein (g)	BG	84.1 ± 5.4	84.2 ± 4.5	82.9 ± 5.2	0.18
	PG	85 ± 7.0	84.3 ± 6.8	83.2 ± 6.0	
Fat (g)	BG	84.5 ± 4.5	85.5 ± 4.2	84.7 ± 5.5	0.65
	PG	86.7 ± 7.2	85.1 ± 6.7	85.2 ± 6.2	

*M* mean; *SD* standard deviations; *PG* placebo group; *BG* betaine group; *MET* Main Effect of Time; Diet Participants’ intake data were analyzed using with repeated measurements ANOVA

#### Monitoring internal training loads

The rating of perceived exertion (RPE) was used to measure internal training loads [35]. Following a cool-down after each session (30 min later), subjects were asked to indicate their perceived exertion on a 0–10-point scale. The RPE was multiplied by the duration of the exercise session to calculate the sRPE [26, 29].

#### Wellness monitoring

Hopper index was used to assess fatigue, recovery and health status of subjects in each session [36, 37]. This index included four subscales: sleep quality, muscle fatigue, delayed onset muscle soreness (DOMS), and stress. Prior to any exercise in order to assess general health indicators, players were asked to rate these variables on a 1 to 7 seven-point Likert scale [37]. This questionnaire was based on Hooper and Mackinnon’s [38].

### Data analysis strategies

Descriptive statistics are reported as mean ± standard deviation. Shapiro-Wilk test and Levene’s test were used to check the normality and homogeneity of variables of data, respectively. Endocrine (GH, IGF-1, testosterone, cortisol, and T/C) and body composition (lean mass, fat mass, BF%, sum of skin folds) variables were assessed with a mixed factorial 2 × 2 analysis of covariance (ANCOVA) with repeated measures. The pre-season value was used as the covariate, time (mid-season and post-season) the within subject factor, and group (BG vs. PG) the between subject factor. Anthropometrics (height, weight, BMI) variables were assessed with a 2 × 3 ANCOVA with repeated measures. The P1 value was used as the covariate, time (mid-season, post-season, follow up) the within subject factor, and group (BG vs. PG) the between subject factor. When a significant time x group interaction was found a one-way repeated-measures analysis of variance (ANOVA) was conducted for each group separately with the Bonferroni correction. If the results of the one-way ANOVA were similar for each group, then the percent changes were computed and assessed with an independent groups t-test. Cohen’s D effect sizes were calculated for each group (P3 – P1 / pooled SD) and defined as trivial (< 0.2), small (≥ 0.2), moderate (≥ 0.5) and large (≥ 0.8). Workload and wellness status data analyzed with repeated measures ANOVA and post hoc analysis was performed according to the method described above. All analyses were conducted with SPSS 22.0 (IBM) and the significant level was set at *p* < 0.05. Also, Excel was used for the exercise pressure and hopper data and the charts were drawn with GraphPad Prism 8.0.1.

## Results

### Monitoring workloads and wellness

**Fig. 1:**
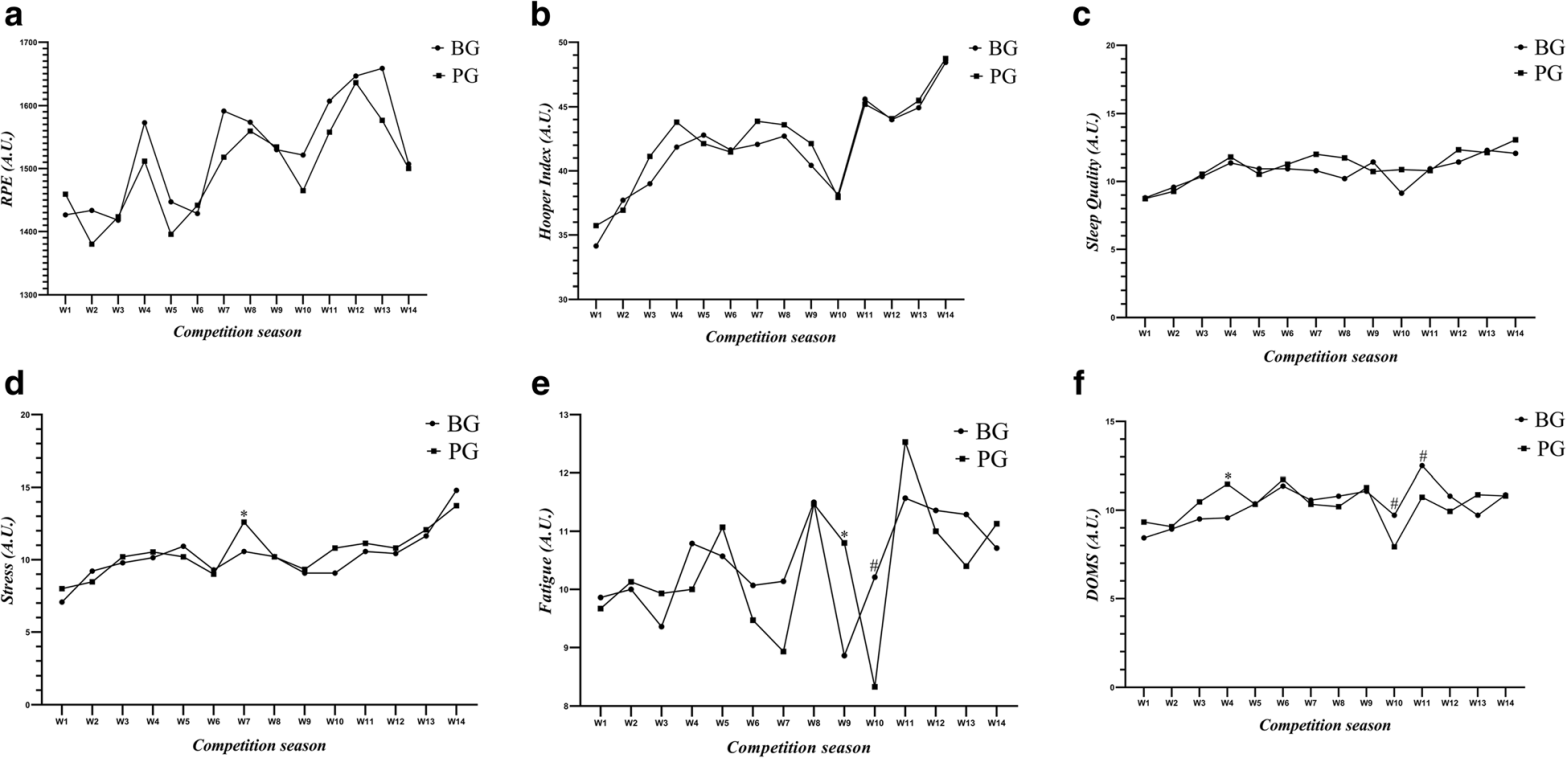
Change in (**a**) sRPE, (**b**) Hooper Index [its subset variables (**c**) sleep, (**d**) stress, (**e**) fatigue and (**f**) DOMS] for 14 weeks in BG and PG. Abbreviation = A.U.: Arbitrary unit; PG: placebo group, BG: betaine group; sRPE: session rating of perceived exertion; DOMS: delayed onset muscle soreness. ∞ Represents a significantly different a main effect of time at that variable during the competition season; * Represents a significantly greater in the PG than the BG; # Represents a significantly greater in the BG than the PG

There was a significant (*p* < 0.001, *f* = 7.741, η_p_^2^ = 0.223) main effect of time but no significant (*p* = 0.96, *f* = 0.424, η_p_^2^ = 0.015) group by time interactions for workload (Fig. 1a). The Hooper index displayed a significant (*p* < 0.001, *f* = 38.964, η_p_^2^ = 0.591) main effect of time but no group by time interaction (*p* = 0.63, *f* = 0.825, η_p_^2^ = 0.030, Fig. 1b). For sleep there were significant (*p* < 0.001, *f* = 11.748, η_p_^2^ = 0.303) main effects of time but not group by time interactions (*p* = 0.09, *f* = 1.558, η_p_^2^ = 0.055, Fig. 1c). There were significant (*p* < 0.001, *f* = 28.529, η_p_^2^ = 0.514) main effects of time and a group by time interaction (*p* = 0.010, *f* = 2.170, η_p_^2^ = 0.074) for stress. Stress values were significantly greater in the PG compared to the BG during week 7 (Fig. 1d). There were significant (*p* < 0.001, *f* = 20.009, η_p_^2^ = 0.237) main effects of time and a group by time interaction (*p* = 0.010, *f* = 2.858, η_p_^2^ = 0.096) for fatigue. Fatigue was significantly greater in the PG than the BG during week 9 but greater in the BG compared to the PG in week 10 (Fig. 1e). There were significant (*p* < 0.001, *f* = 10.404, η_p_^2^ = 0.278) main effects of time and a group by time interaction (*p* < 0.001, *f* = 3.566, η_p_^2^ = 0.117) for DOMS. DOMS was significantly greater in the PG in week 4 but greater in the BG in weeks 10 and 11 (Fig. 1f).

### Endocrine markers

There were no significant (*p* = 0.84, *f* = 0.044, η_p_^2^ = 0.002) main effects of time or group by time interaction (*p* = 0.68, *f* = 0.169, η_p_^2^ = 0.006) for changes in IGF-1. There were no significant (*p* = 0.28, *f* = 1.230, η_p_^2^ = 0.045) main effects of time or group by time interaction (*p* = 0.32, *f* = 1.045, η_p_^2^ = 0.039) for changes in cortisol. There were no significant group by time interactions for GH (*p* = 0.16, *f* = 2.124, η_p_^2^ = 0.076), however, there was a main effect of time *(p* = 0.04, *f* = 4.891, η_p_^2^ = 0.158).

There were no significant (*p* = 0.61, *f* = 0.261, η_p_^2^ = 0.010) main effects of time for testosterone, but there was a significant group by time interaction (*p* = 0.001, *f* = 15.140, η_p_^2^ = 0.368). Post hoc analysis revealed testosterone was significantly (*p* < 0.001) greater at P2 and P3 compared to P1 for BG, but for PG was significantly (*p* < 0.001) less at P3 compared to P2 and showed a non-significant (*p* = 0.070) trend to be less at P3 compared to P1. There were no significant (*p* = 0.42, *f* = 0.669, η_p_^2^ = 0.025) main effects of time for the T/C ratio, but there was a significant group by time interaction (*p* = 0.005, *f* = 9.379, η_p_^2^ = 0.265). Post hoc analysis revealed the T/C ratio significantly increased from P1 to P2 and P3 (*p* < 0.001) and P2 to P3 (*p* = 0.048) in the BG whereas there were no significant differences between P1, P2, or P3 in the PG (Table 3).

**Table 3:** Changes in body composition and endocrine markers between pre-post season

Variables	Groups	Pre-Season	Mid- Season	Post- Season	Mean Pre-Post Season (95% CI)		Pre-Post Season % Change	Effect Size
		M ± SD	M ± SD	M ± SD	Lower	Upper		Cohens D
*Body composition*								
Body Fat %	BG	8.7 ± 2.7	7.8 ± 2.4	7.8 ± 2.4	−13.1	−6.8	−10	−0.3 S
	PG	9.2 ± 3.5	8.6 ± 3.2	8.4 ± 3.1	−9.5	−6.5	−8	−0.2 S
Body Fat (kg)	BG	5.1 ± 1.6	4.6 ± 1.4	4.7 ± 1.5	−12.1	−6.6	−9.3	−0.3 S
	PG	6.2 ± 2.7	5.8 ± 2.6	5.7 ± 2.5	− 9	−5.2	−7.1	−0.2 T
LBM (kg)	BG	54.0 ± 4.6	54.0 ± 4.6	55.0 ± 4.9	0.4	3.1	1.7	0.2 S
	PG	59.6 ± 5.4	59.9 ± 5.4	60.7 ± 5.1	0.9	2.8	1.8	0.2 S
Sum of 7-SS (mm)	BG	71.6 ± 17.9	65.9 ± 15.7	65.1 ± 15.1	−10.2	−7.4	−8.8	−0.4 S
	PG	75.4 ± 23.7	71.5 ± 21.9	70.2 ± 21.1	−7.8	−5.1	−6.5	−0.2 S
*Endocrine Markers*								
IGF-1 (ng/mL)	BG	401 ± 98.6	477.3 ± 79.4	496.2 ± 80.6	14.6	42.1	28.4	1.1 L
	PG	326.8 ± 64.0	353.5 ± 68.4	366.4 ± 67.3	6.6	19.2	12.9	0.6 M
GH (ng/mL)	BG	1.3 ± 0.4	2.36 ± 0.9	4.5 ± 3.2	130	300.6	215.3	1.4 L
	PG	1.3 ± 0.4	2.0 ± 0.7	2.8 ± 1.9	54.4	191	122.7	1.1 L
Testosterone (ng/mL)	BG	3.3 ± 1.4	5.4 ± 2.1^€^	5.9 ± 1.9^*^	49.3	186	117.6	1.6 L
	PG	3.6 ± 1.0	4.1 ± 0.6	2.9 ± 0.7^#^	−30.7	0.8	−14.9	−0.8 L
Cortisol (μg/dL)	BG	15.1 ± 2.1	12.7 ± 2.4	11.8 ± 2.1	−28.7	− 13.5	−21.1	−1.6 L
	PG	13.5 ± 1.8	16.0 ± 2.5	13.6 ± 3.5	−12.7	14.8	1.1	≤0.001 T
T/C	BG	0.2 ± 0.1	0.5 ± 0.2^€^	0.4 ± 0.2^*^	72.8	127	199.79	1.8 L
	PG	0.2 ± 0.1	0.3 ± 0.1	0.3 ± 0.1	−29.3	17.8	−11.51	−0.6 M

*BG* Betaine Group; *PG* Placebo Group; *LBM* Lean body mass; *IGF-1* Insulin like growth factor-1; *T/C* testosterone to cortisol ratio; *GH* Growth hormone; Sum of 7-SS: sum 7 skinfolds of the site (i.e., chest, abdominal, thigh, triceps, subscapular, suprailiac and midaxillary); *P1* Pre-Season; *P2* Mid- Season; *P3* Post- Season; *T* Trivial; *S* Small; *M* Moderate; *L* Large

^€^Represents a statistically significant difference compared to P1-P2 (*p* < 0.05); ^#^Represents a statistically significant difference compared to P2-P3 (*p* < 0.05); ^*^Represents a statistically significant difference compared to P1-P3 (*p* < 0.05)

### Body composition and anthropometrics

There were no significant (*p* = 0.52, *f* = 0.431, η_p_^2^ = 0.016) main effects of time or group by time interaction (*p* = 0.24, *f* = 1.457 η_p_^2^ = 0.053) for body fat percentage. There were no significant (*p* = 0.88, *f* = 0.024, η_p_^2^ = 0.001) main effects of time or group by time interaction (*p* = 0.12, *f* = 2.641 η_p_^2^ = 0.092) for changes in fat mass. There were no significant group by time interactions for change in sum of skinfolds (*p* = 0.19, *f* = 1.844, *η*_*p*_^*2*^ = 0.066), however, there were significant a main effect of time (*p* = 0.02, *f* = 6.472, *η*_*p*_^*2*^ = 0.199). There were no significant (*p* = 0.30, *f* = 1.134, η_p_^2^ = 0.042) main effects of time or group by time interaction (*p* = 0.97, *f* = 0.002 η_p_^2^ = 0.001) for changes in lean body mass (Table 3).

There were no significant main effects across time for height (*p* = 0.44, *f* = 0.839, η_p_^2^ = 0.031). There was a significant group by time interaction for height (*p* = 0.002, *f* = 7.028, η_p_^2^ = 0.213). Post hoc analysis revealed height was significantly greater at follow up (*p* < 0.001) versus all other time points for both BG and PG. When comparing the percent change from P3 to follow up, the results of an independent groups t-test revealed a significant difference between BG and PG (*p* = 0.017). Height in the BG increased by 5.2% (95% CI: 2.6–7.80%) compared to 3.2% (95% CI: 2.35–4.05%) in PG. There were no significant main effects across time for weight (*p* = 0.32, *f* = 1.151, η_p_^2^ = 0.042). There was a significant group by time interaction for weight (*p* = 0.047, *f* = 3.242, η_p_^2^ = 0.111). For betaine, post hoc analysis revealed weight was significantly less at P2 versus P1 (*p* = 0.039), returned to P1 values at P3, and was significantly greater at follow up versus all other time points (*p* < 0.001). For PG, weight was significantly greater at follow up versus all other time points (*p* < 0.001), but there were no significant differences across the competitive season. When comparing the percent change from P3 to follow up, the results of an independent groups t-test revealed a significant difference between BG and PG (*p* = 0.009). Weight in the BG increased by 13.3% (95% CI: 9.40–17.20%) compared to 7.0% (95% CI: 4.78–9.22%) in PG. For BMI there were significant main effects of time (*p* < 0.001, *f* = 9.474, η_p_^2^ = 0.267), but no significant interactions (*p* = 0.73, *f* = 0.315, η_p_^2^ = 0.012). BMI at mid and P3 were significantly less than P1, but not significantly less than follow up. Height, weight, and BMI can be found in supplementary Fig. 1a-1c.

## Discussion

The aim of this study was to investigate the effects of betaine supplementation during a 14-week competitive season in professional youth soccer players. We hypothesized that betaine supplementation would reduce hormonal indices of NFO and improve body composition compared to PG. Although lean mass increased and fat mass decreased during the study, there was no significant differences between groups. Similarly, there were no differences between groups in GH nor IGF-1. On the other hand, testosterone increased in the BG and decreased in the PG, and the T/C increased in the BG but did not change in PG, despite both groups performing a similar workload and reporting similar Hooper indexes.

Despite small improvements in body composition in both groups, betaine supplementation did not promote a greater reduction in fat mass nor increase in lean mass. These findings are consistent with Schwab et al. [39] where no significant differences after 12 weeks of betaine administration were found. On the other hand, Cholewa et al. examined 2.5 g of betaine supplementation for 6 weeks on individuals engaged in resistance training. The results showed that supplementation with training had a significant effect on body composition with a reduction in BF% and increase in lean mass [15]. Cholewa et al. conducted another study on the effect of betaine consumption in conjunction with resistance training on body composition in untrained college women. They reported only significant inter-group differences for changes in fat mass and BF%, but not lean mass [40]. Discrepancies in results between these studies and ours may be attributable to differences in age, lack of resistance training, the voluminous aerobic volume load of the season, and/or insufficient protein intake.

Training to reach peak physical performance requires a balance between workload volume and recovery, and when fatigue exceeds recovery NFO ensues. Endocrine hormones may be routinely monitored and used as biomarkers of physiological alterations that may predict the onset of NFO or over training during a soccer season [11]. Several studies have reported a decrease in testosterone following a soccer season [12, 41], and changes to the hypothalamic-pituitary axis (HPA), in particular testosterone and cortisol [42], and a change in the T/C of greater than 30% are associated with the development of NFO [12].

In the present study, testosterone values were distinctly different between groups, increasing in the BG over the course of the season but decreasing in the PG. Although when controlling for pre-season values of cortisol there were no statistically significant differences between groups, large effect sizes (d = − 1.57) for a reduction in cortisol were found for the BG but not PG, and the T/C increased in the BG only. These changes in steroid hormone concentrations seemingly occurred despite no differences between groups in workload, sleep, stress, or nutritional status. These results suggest that betaine supplementation may be a useful nutritional adjunct to delay the onset of NFO.

The mechanisms whereby betaine may influence the HPA directly or downstream to affect testosterone and cortisol concentrations are not fully understood. Although there is no data in humans, betaine is highly concentrated in testicular tissue (i.e., 2.63 μmol/g tissue in testes vs. 0.18 μmol/g tissue in skeletal muscle) and a direct correlation between plasma betaine values and testicular concentrations exist [43] {Slow, 2009 #121}. In mice exposed to arsenic, betaine supplementation prevented a decrease in plasma testosterone and dihydrotestosterone, likely by maintaining testicular glutathione and catalase, and preventing oxidative damage in Leydig cells [44].

In regards to cortisol, betaine concentrations were found to be negatively correlated to cortisol concentrations in elderly [45], and Apicella et al. reported a decrease in cortisol following 2 weeks of betaine supplementation in healthy young adults [21]. Although the potential mechanisms whereby betaine may affect cortisol concentrations are not fully understood, it is possible that betaine’s role in methylation metabolism may be involved. In middle-aged adults, plasma homocysteine concentrations were found to be positively correlated with plasma cortisol [46], and occupations of greater stress demonstrate higher plasma homocysteine concentrations than those of low stress [47]. Homocysteine levels have been shown to increase by an average of 3.8% following a 15-min mentally stressful activity [48], and both acute and chronic aerobic training increases homocysteine concentrations, with the volume of workload contributing most to these increases [49]. In the liver, catabolism of betaine via the betaine homocysteine methyl-transferase (BHMT) enzyme results in the transmethylation of homocysteine to methionine, and betaine ingestion has been consistently shown to reduce plasma homocysteine levels [50]. Hepatic BHMT activity is also increased as a result of corticosteroid secretion [51]. Whether elevated homocysteine is a stimulus for increased corticosteroid secretion, or there is an increase in corticosteroid secretion to stimulate BHMT in order to reduce homocysteine levels is currently unknown. However, by reducing plasma homocysteine, betaine may play a role in maintaining lower resting cortisol concentrations.

Very few studies have examined the long-term impact of soccer training on GH secretions in youth athletes. In a study by Mejri et al. GH secretion decreased during the competition season in U19 players, but there were no changes in IGF-1 secretion [52]. In contrast, Hammami et al. performed a 2-year study on both the U17 national soccer team preparing for the African championship and a non-athlete control group. The athlete group had higher hormonal secretion than the control group at all five evaluation periods during the 2 year period [53]. Wheldon et al. suggests the reasons for the inconsistency in the GH response to competitive sports depends on intensity, duration, type of activity, gender of subjects, age of the athlete, environmental factors, and nutrition [54].

In humans 2 weeks of betaine supplementation increased IGF-1 and tended to increase GH [21]. Several animal studies have demonstrated an increase in GH concentrations and GH pulsatile secretions with betaine ingestion [55], likely due to an increase in hypothalamic betaine tissue concentrations [56] which may lead to an increase in GHRH gene expression [57]. However, betaine supplementation did not result in an increase in GH or IGF-1 in the present study. Although betaine supplementation increases IGF-1 and IGF-1 receptor signaling in vitro [22], its effects on growth factors in humans remain unclear.

Another purpose of this study was to examine the effects of 14 weeks of betaine supplementation on physical development. The average height of 16 and 17-year-old males has been increasing approximately by 2 to 3 cm per decade in Iran since 1991 (the year 1369 on the Solar Hijri calendar) [53, 58]. In the present study, there were greater increases in height and weight between P3 and follow-up in the BG compared to the PG. While we cannot attribute these greater indices of growth to betaine supplementation, it does suggest that betaine supplementation did not negatively affect growth and development.

This study is not without limitations that need to be considered. First, we could not control the participants’ nutrition on all study days. Second, we did not have access to tools to measure external workload, such as Global Positioning System (GPS) for distance traveled. Finally, the adolescent population in this study and adults used to formulate the Jackson-Pollock and Brozek equations are not the same, which may introduce error into the calculation of body fat percentage. To account for this potential error, we have also reported sum of skin folds.

## Conclusions

Fourteen weeks of betaine supplementation prevented the reduction in testosterone associated with the physical stressors of a soccer-season and increased the T/C in youth professional soccer players. These changes in steroid hormones suggest that betaine supplementation may be a useful nutritional strategy to offset the development of NFO. The results of the one-year follow-up suggest that betaine supplementation does not negatively impact growth, however, more research is necessary to determine if the increases in anabolic hormones are sustainable for longer than 14 weeks of supplementation, and if a longer supplementation period would translate to meaningful physiological adaptations.

## Supplementary Information

**Additional file 1.**

## Data Availability

The datasets used and/or analyzed during the current study are available from the corresponding author on reasonable request.

## Abbreviations

NFO: Nonfunctional over-reaching state
T/C: Testosterone to cortisol ratio
GH: Growth hormone
IGF-1: Insulin-like growth factor-1
IL-1ß: Interlukin-1 beta
LBM: Lean body mass
BF: Body fat
RPE: Rating of perceived exertion
ICMA: Chemiluminescence method
BG: Betaine group
PG: Placebo group
P1: Pre-season
P2: Mid-season
P3: Post-season
F: Follow up
DOMS: Delayed onset muscle soreness
