# Supplementary figures and images for: The effects of 14-week betaine supplementation on endocrine markers, body composition and anthropometrics in professional youth soccer players: a double blind, randomized, placebo-controlled trial
Source: J Int Soc Sports Nutr. 2021 Mar 4;18:20. doi: 10.1186/s12970-021-00417-5 (PMC7934563; doi:10.1186/s12970-021-00417-5)

Figure 2A

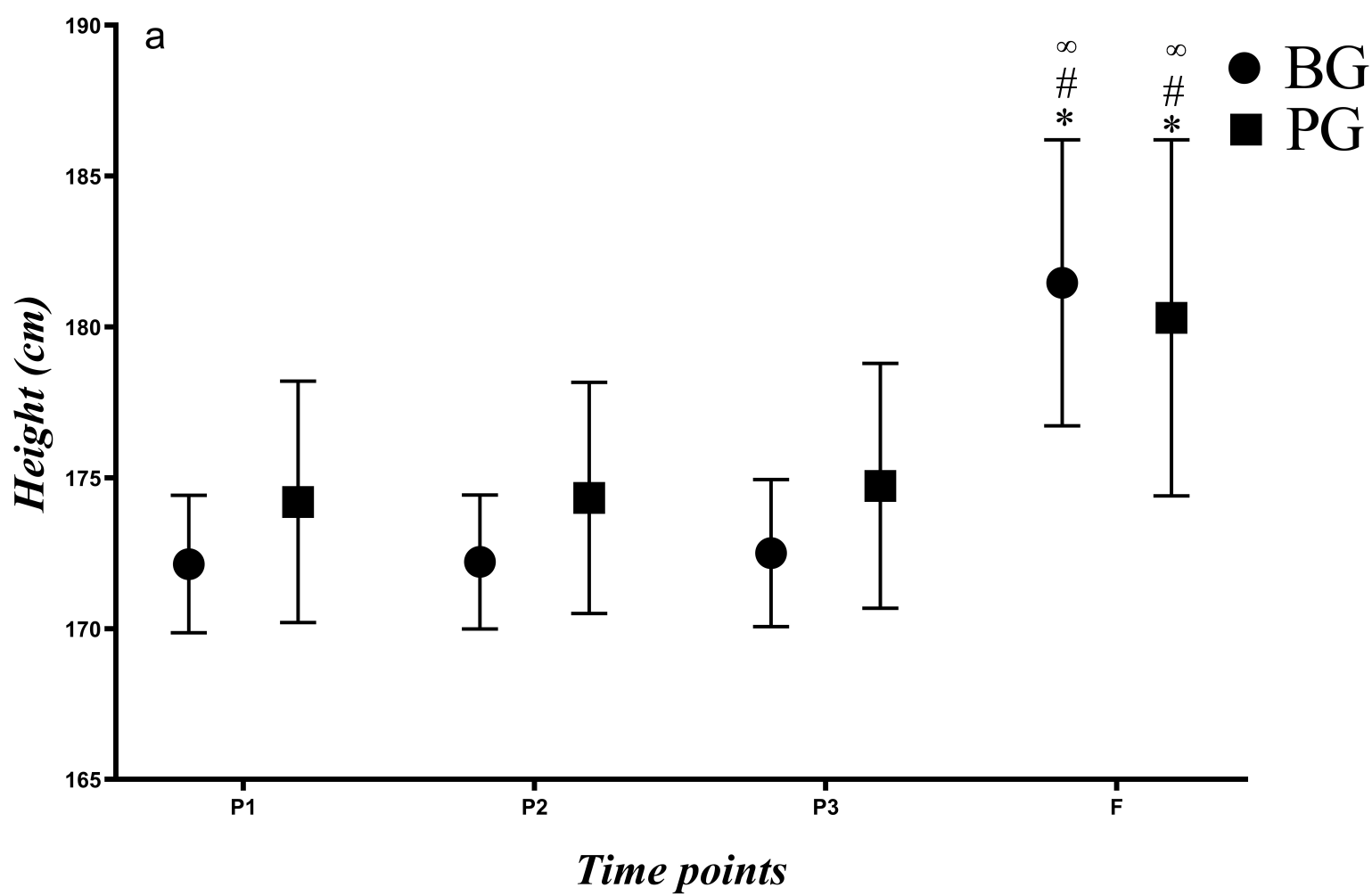

Figure 2B

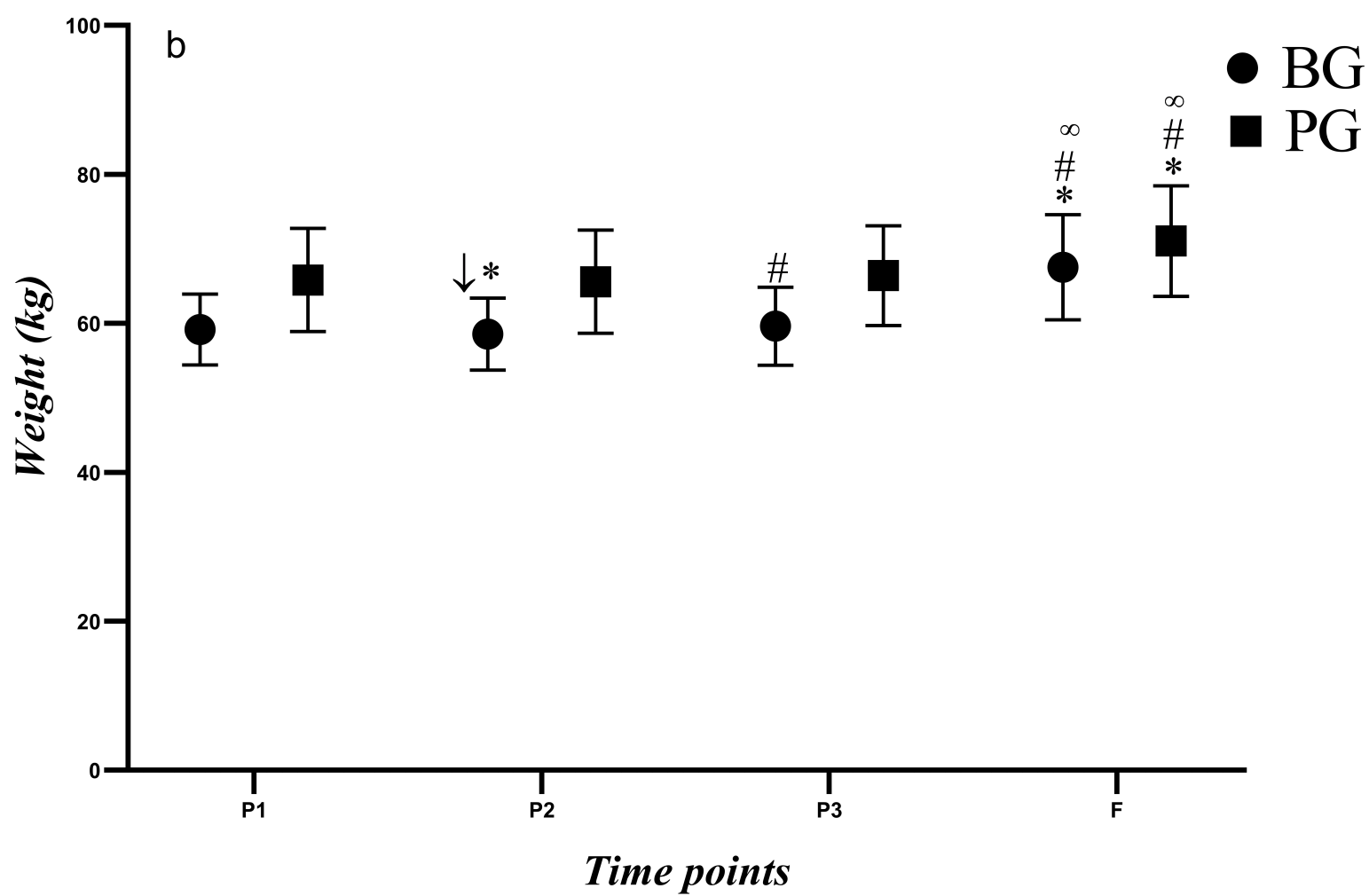

Figure 2C

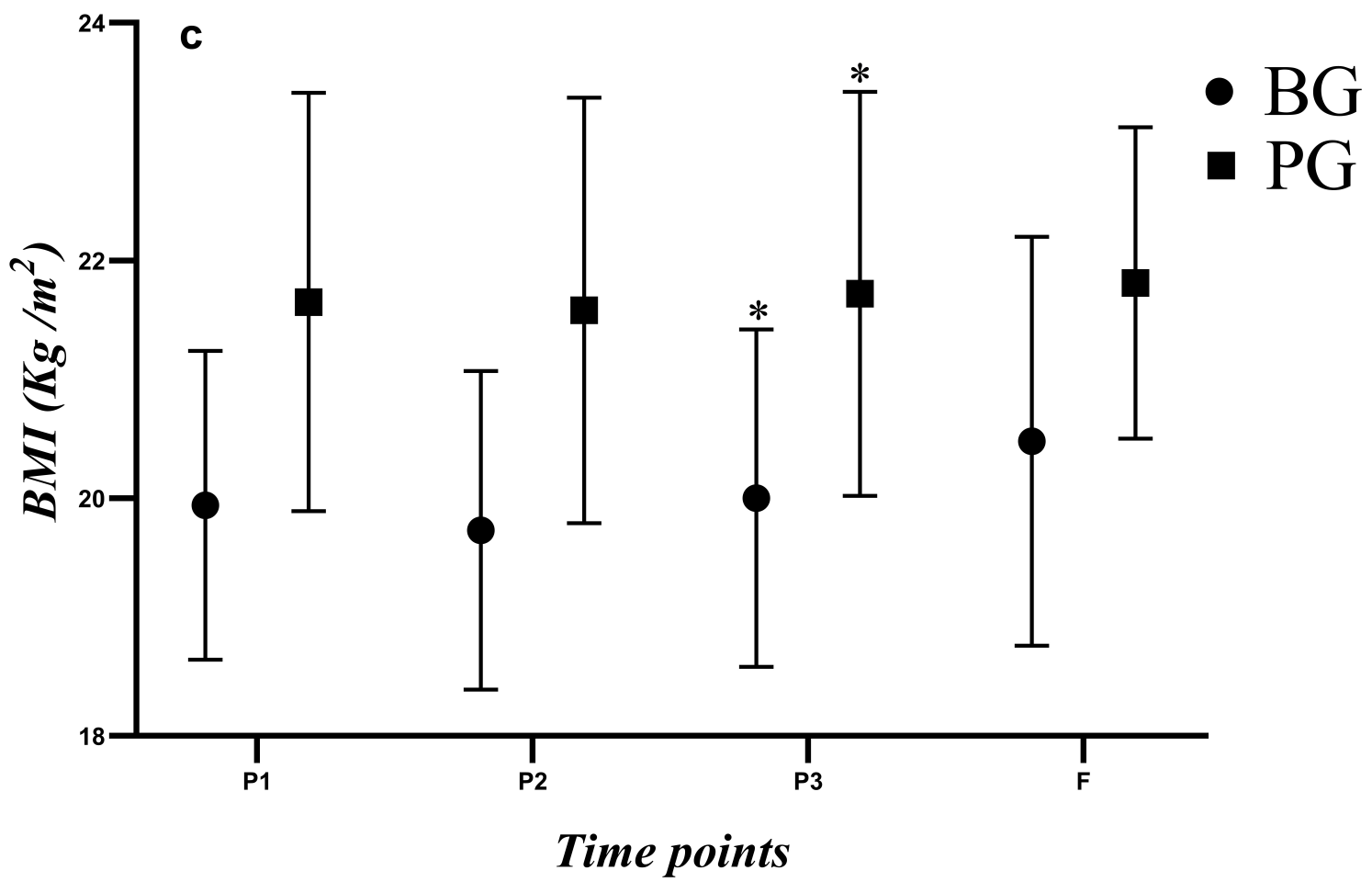

Supplement: Supplementary file 1 — Additional file 1. [file 12970_2021_417_MOESM1_ESM.pdf]
